# Eco-Friendly Cavity-Containing Iron Oxides Prepared by Mild Routes as Very Efficient Catalysts for the Total Oxidation of VOCs

**DOI:** 10.3390/ma11081387

**Published:** 2018-08-09

**Authors:** Rut Sanchis, Daniel Alonso-Domínguez, Ana Dejoz, María Pilar Pico, Inmaculada Álvarez-Serrano, Tomás García, María Luisa López, Benjamín Solsona

**Affiliations:** 1 Departament d’Enginyeria Química, ETSE, Universitat de València, Av. Universitat, 46100 Burjassot, Valencia, Spain; rut.sanchis@uv.es (R.S.); ana.m.dejoz@uv.es (A.D.); 2 Departamento de Química Inorgánica, Facultad de Ciencias Químicas, Universidad Complutense de Madrid, 28040 Madrid, Spain; daniel.alonso@csic.es (D.A.-D.); ias@quim.ucm.es (I.Á.-S.); 3 Sepiolsa, Avda. del Acero, 14-16, Pol. UP-1 (Miralcampo), 19200 Azuqueca de Henares, Spain; maria.pico@sepiolsa.com; 4 Instituto de Carboquímica (ICB-CSIC), C/Miguel Luesma 4, 50018 Zaragoza, Spain; tomas@icb.csic.es

**Keywords:** volatile organic compounds (VOC), iron oxide, mild preparation routes, total oxidation, cavities

## Abstract

Iron oxides (FeOx) are non-toxic, non-expensive and environmentally friendly compounds, which makes them good candidates for many industrial applications, among them catalysis. In the present article five catalysts based on FeOx were synthesized by mild routes: hydrothermal in subcritical and supercritical conditions (Fe-HT, Few200, Few450) and solvothermal (Fe-ST1 and Fe-ST2). The catalytic activity of these catalysts was studied for the total oxidation of toluene using very demanding conditions with high space velocities and including water and CO_2_ in the feed. The samples were characterized by X-ray diffraction (XRD), scanning and high-resolution transmission electron microscopy (SEM and HRTEM), X-ray photoelectron spectroscopy (XPS) and nitrogen adsorption-desorption isotherms. It was observed that the most active catalyst was a cavity-containing porous sample prepared by a solvothermal method with a relatively high surface area (55 m^2^ g^−1^) and constituted by flower-like aggregates with open cavities at the catalyst surface. This catalyst displayed superior performance (100% of toluene conversion at 325 °C using highly demanding conditions) and this performance can be maintained for several catalytic cycles. Interestingly, the porous iron oxides present not only a higher catalytic activity than the non-porous but also a higher specific activity per surface area. The high activity of this catalyst has been related to the possible synergistic effect of compositional, structural and microstructural features emphasizing the role of the surface area, the crystalline phase present, and the properties of the surface.

## 1. Introduction

The removal of volatile organic compounds (VOCs) from the environment is an essential problem that must be addressed urgently. Accordingly, there are several technologies developed which are currently used at an industrial level. Among the technologies which eliminate VOCs without destroying them, we can mention adsorption and absorption; these two techniques are not very complex and have low energy consumption. However, the amount of VOC removed is on many occasions insufficient. Among the technologies that destroy VOCs, thermal combustion is widely employed. Unfortunately, the temperature required is high and undesired toxic compounds are often produced. The use of catalysts (catalytic oxidation) has been shown to be an optimal option as it minimizes the VOC concentration in the exhaust gases, requires lower temperatures than thermal combustion, and the emission of toxic compounds is usually low [1].

Although most catalysts usually employed are based on noble metals [2,3], in the last few decades, non-noble transition metal oxides and oxy-hydroxides have been investigated as catalysts in reactions in which VOCs are destroyed by total oxidation processes [4,5,6]. Among transition metal oxides, those of manganese and cobalt have shown an excellent catalytic performance, in some cases comparable to noble metal-derived catalysts [7,8]. Iron oxide catalysts, if conveniently prepared [9,10,11,12,13], can also present high catalytic activity, being of great interest environmentally, as they are abundant and non-toxic. Thus, bare ferrite-type iron oxides or those impregnated on clays [14,15], obtained from different sources with positive environmental implications, have been studied [16]. The use of simple supported Fe_2_O_3_/Al_2_O_3_ catalysts has also been studied, displaying reasonably high activity in the total oxidation of toluene [17]. In another work, our group showed that the use of porous clay heterostructures as supports leads to highly active FeOx-species, displaying activities two orders of magnitude higher than those obtained when pure silica is used as a support [18]. This performance was assigned to the presence of dispersed iron species on the support which could easily activate the substrate. Bulk iron (III) oxides [19] have also been studied as catalysts for toluene oxidation. It has been reported that materials with mesoporous morphology are highly active. Interestingly, the main factor affecting catalytic activity is the surface area. Hence, the iron oxide leading to the highest reactivity is that synthesized by a nanocasting method, which presents a specific surface area of 208 m^2^ g^−1^. On the other hand, it has also been reported that one way to promote the efficiency in eliminating toluene by using iron mixed oxides is to employ a microwave furnace system [20].

Different synthetic routes, e.g., solid state, hydrothermal, molecular sieve techniques, have been explored to optimize the catalytic performance taking into account environmental issues, cost, feasibility and stability considerations. Thus, for example molecular sieve processes lead to iron oxide-based catalysts with high stability. However, a “rapid” route (a solvothermal process with a further calcination at 500 °C) has been revealed as highly appropriate bearing in mind aspects such as simplicity, low-cost, reaction rate and controllability [21]. Besides, hydrolysis-driven redox reactions have also been explored for obtaining iron oxides highly active as catalysts for toluene oxidation and with high tolerance to moisture [11].

As mentioned above, high surface area has been reported to be one of the most relevant parameters leading to high catalytic activity iron oxides in the toluene total oxidation. If these porous iron oxides could be synthesized by new mild, low-cost and innocuous methods leading to iron oxides with interesting morphologies it would mean a step forward. Accordingly, this paper reports the synthesis and characterization of different iron oxide samples obtained by facile, low-cost and environmentally friendly processes: hydrothermal (batch and continuous regimes) in supercritical and subcritical conditions, and solvothermal procedures, which were further tested for the total oxidation of toluene.

The choice of toluene as a VOC is due to the fact that aromatic hydrocarbons are one of the most toxic subset of the VOCs and, therefore, its removal presents a paramount importance. Moreover, we have studied reaction conditions in which the space velocity is very high (Gas Hourly Space Velocity, GHSV = 300,000 h^−1^) and the toluene concentration low (1000 ppm) in order to simulate quasi-industrial conditions. The effect of the presence of water and CO_2_ in the feed has also been studied.

## 2. Materials and Methods

### 2.1. Preparation of Iron Oxide Catalysts

Few200 and Few450 iron oxide samples were prepared from 0.4 M iron (II) acetate (99%, Sigma-Aldrich, St. Louis, MO, USA) in supercritical water, employing the continuous hydrothermal experimental home-made set-up described previously [22,23]. The precursor solutions were freshly prepared, titrated by permanganometry and treated with iron nails for preserving the iron oxidation state. Distilled water was preheated and pressurized up to supercritical conditions. Then, this pretreated water and the iron (II) acetate solution were introduced in the system using high-pressure pumps at 7:3 flow ratio. The reactor temperature was maintained at 200 °C (Few200) or 450 °C (Few450) in each case. Details can be found elsewhere [23]. In these conditions, majority spinel-type phases (maghemite and magnetite) are obtained, coexisting with a small percentage of hydrated oxides. They were further treated under an inert atmosphere up to 400 °C for 3 h, showing a stable behavior. From routine thermogravimetric experiments and infrared (IR) spectroscopy curves (Supporting Information, Figure S1), the total decomposition of possible FeOOH-type phases at that temperature was observed. Therefore hydroxide-type phases can be assumed to be absent in the prepared catalyst.

The Fe-HT sample was obtained from 0.1 M iron (II) acetate, Fe(C_2_H_3_O_2_)_2_ (99%, Sigma-Aldrich, St. Louis, MO, USA), aqueous solution. The solution was further introduced in an autoclave and treated at 180 °C for 12 h. The precipitate obtained was then filtered through a polymeric membrane with an average pore size of 200 nm (polyethersulphone from Sartorius) and finally dried in a stove at 100 °C. Finally, the sample was treated under an inert atmosphere (He flow) at 400 °C for 3 h. The iron oxide phase observed was hematite.

The Fe-ST1 sample was obtained similarly to Fe-HT sample but using a solution of ethylene glycol instead of an aqueous solution. The solution was autoclaved at 180 °C, and the precipitate obtained was filtered and then dried overnight at 100 °C. Finally, the catalyst was treated in He at 400 °C for 3 h. The iron oxide obtained was mainly a spinel-type phase.

The Fe-ST2 sample was obtained similarly to Fe-HT and Fe-ST samples but using a 0.075 M solution of polyethylene glycol (PEG) in ethylene glycol. The solution was autoclaved at 180 °C, and the precipitate obtained was filtered and then dried overnight at 100 °C. Finally, the catalyst was treated in He at 400 °C for 3 h.

For comparative purpose, two iron oxides were tested in the same conditions: (i) commercial iron oxide (Fe_2_O_3_) from Panreac subsequently calcined for 4 h at 500 °C which presents a surface area of 3.3 m^2^ g^−1^; and (ii) a high surface area (208 m^2^ g^−1^) mesoporous Fe_2_O_3_ synthesized by a nanocasting method [18,19].

### 2.2. Physicochemical Characteristics of the Catalysts

X-ray powder diffraction (XRD) patterns were registered at room temperature with a PANanalytical X’PERT POWDER diffractometer (Malvern Panalytical Ltd., Malvern, UK) using Cu (Kα) radiation with λ = 1.5418 Å.

For the electron microscopy (EM) analyses, two microscopes were employed. Scanning electron microscopy (SEM) was performed by using a JEOL JSM 6335F (The McCrone Group, Westmont, IL, USA), with resolution of 12 Å. Samples were metallized by covering with Au. High-resolution transmission electron microscopy (HRTEM) results were obtained using a JEOL JEM 3000F (300 kV) microscope (The McCrone Group, Westmont, IL, USA), inTransmission Electron Microscopy (TEM) and Scanning Transmission Electron Microscopy (STEM) modes. Samples were prepared by crushing the powders under n-butanol and dispersing them over copper grids covered with a holed carbon film.

Nitrogen adsorption measurements were performed on a Micromeritics ASAP 2010 physisorption analyzer (Micromeritics, Norcross, GA, USA) at −196 °C. Brunauer–Emmett–Teller (BET) and Barret–Joyner–Halenda (BJH) methods were used for the surface area and pore size distribution determination using N_2_ adsorption data. Samples were previously outgassed for at least 4 h at 120 °C.

XPS (X-ray photoemission) measurements were conducted using ESCALAB 210 multi-analysis system (Thermo Fisher Scientific, Waltham, MA, USA) (pressure 1.0 × 10^−10^ mbar). Photoelectrons were excited with the MgKα line. The hemispheric photoelectron analyzer (Thermo Fisher Scientific, Waltham, MA, USA) worked with a pass energy of 20 eV. C 1s core level of adventitious carbon was taken as a reference. Its binding energy (BE) was assigned to be 284.8 eV.

### 2.3. Catalytic Measurements

The catalytic activity in the total oxidation of toluene was determined using a fixed bed laboratory micro-reactor. The reaction conditions were very demanding fixing in all cases the space velocity in 300,000 h^−1^. Most of the catalytic assays were done using as the reactant mixture 1000 vppm of toluene in synthetic air (20% O_2_ and 80% He). In some selected experiments, 4 or 10% water steam and/or 0.2% CO_2_ were also used. Reactants and reaction products were analyzed by gas chromatography using both thermal conductivity and flame ionization as detectors. Two chromatographic columns were used: (i) Porapak Q (for CO_2_, water and hydrocarbons) and (ii) Molecular Sieve 5A (to separate O_2_, N_2_ and possible traces of CO). The range of reaction temperatures studied was 100–400 °C.

The toluene conversion was determined in two ways: (i) by the difference between the inlet and outlet toluene concentration, and (ii) by the amount of CO_2_ and toluene after reaction. These two procedures led us to adjust the carbon balance with an accuracy of ±10% at low toluene concentration, whilst the accuracy greatly improved up to ±3% at toluene conversions higher than 20%. Two analyses were undertaken at each reaction temperature once steady state activity was achieved (30 min) and the results were averaged.

Blank runs were carried out without catalysts (empty reactor) up to 500 °C, showing negligible conversion.

## 3. Results

### 3.1. Catalytic Results

Iron oxide catalysts were tested in the total oxidation of toluene (1000 ppm of toluene in synthetic air) using high space velocities. The only reaction product observed was CO_2_. Neither partially oxygenated compounds nor CO were detected in any experiment. As CO_2_ is observed according to the drop of the toluene area, we can conclude that this is the result of the toluene transformation solely into carbon dioxide.

**Figure 1:**
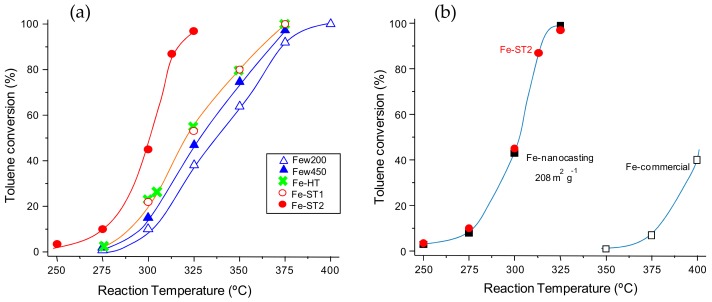
Evolution of the toluene conversion with the reaction temperature for the differently prepared FeOx catalysts: (**a**) comparison of the iron oxides synthesized in the present work, (**b**) comparison between Fe-ST2 catalyst and representative catalysts Fe-com and Fe-nano. Note: Gas Hourly Space Velocity (GHSV) = 300,000 h^−1^ and remaining reaction conditions detailed in the text. Symbols: ∆ Few200, ▲ Few450, × Fe-HT, ○ Fe-ST1, ● Fe-ST2, □ Fe-com, ■ Fe-nano.

Figure 1a shows the variation of the toluene conversion with the reaction temperature for the iron oxide catalysts synthesized. It can be observed that the most active catalyst was that prepared by a solvothermal method in the presence of polyethylene glycol (Fe-ST2). Indeed, the use of a Fe-ST2 catalyst enables initialization of the transformation of toluene at temperatures lower than 250 °C and to reach 100% conversion at 325 °C. In contrast, the other catalysts achieved total conversion at temperatures of 375 °C and higher. The least active sample was that prepared in supercritical water at 200 °C (Few200) whereas the other samples prepared by different procedures (supercritical water Few450, solvothermal Fe-ST1 or hydrothermal Fe-HT) presented an intermediate catalytic activity.

For comparison, in Figure 1b the most active catalyst, Fe-ST2, is compared against two representative catalysts: a commercial iron oxide (Fe-com) and also with a very high surface area mesoporous iron oxide prepared by nanocasting (Fe-nano, 208 m^2^ g^−1^). As can be seen, the Fe-ST2 catalyst presents a light-off curve very similar to that of the nanocasting sample, in spite of the fact that the surface area is remarkably lower in the Fe-ST2 catalyst (55 m^2^ g^−1^). Therefore, the intrinsic reactivity of the iron sites of Fe-ST2 is higher than those of Fe-nano as the areal rate is remarkably higher. Moreover, if compared to the commercial iron oxide, the catalytic activity is several orders of magnitude higher in Fe-ST2. Therefore, not only the catalytic activity is higher but also the activity normalized per surface area. Thus, in order to achieve similar conversion the commercial catalyst requires a temperature ca. 100 °C higher than Fe-ST2.

**Figure 2:**
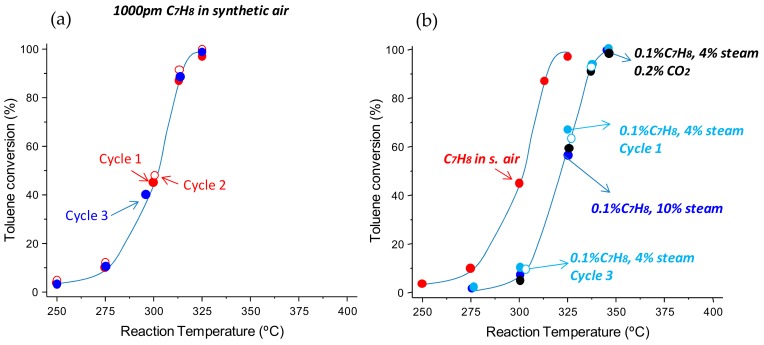
Evolution of the toluene conversion with the reaction temperature for Fe-ST2 catalyst: (**a**) stability after 3 catalytic cycles, (**b**) effect of the presence of water steam and CO_2_ on the catalytic performance. GHSV = 300,000 h^−1^ and remaining reaction conditions detailed in the text.

The stability of the most active catalysts were also studied (Figure 2a). Thus, three cycles were undertaken on Fe-ST2 and Fe-HT (not shown). The three catalytic cycles were carried out over three consecutive days, keeping the sample overnight in the reaction system with a flow of helium. Interestingly, no appreciable differences in conversion were observed in the experiments conducted. Therefore, the Fe-HT catalyst was also demonstrated to be highly stable. As in industrial effluents the VOCs do not flow alone with air, and water and/or carbon dioxide have been fed together with toluene (Figure 2b). Unfortunately, the presence of water is deleterious as, for a fixed reaction temperature, the toluene conversion drops. Thus, after adding 4–10% of water the light-off curves are shifted ca. 25 °C towards higher temperatures. Interestingly, the negative effect of water does not lead to a progressive fall in consecutive cycles, maintaining a stable performance after 3 cycles. The effect of the addition of low concentrations of CO_2_ together with water was also studied, showing scarce effects on the catalytic activity compared to the water-only experiments.

### 3.2. Characterization Results

Some physicochemical characteristics of the iron oxide catalysts synthesized in the present work are shown in Table 1.

**Figure 3:**
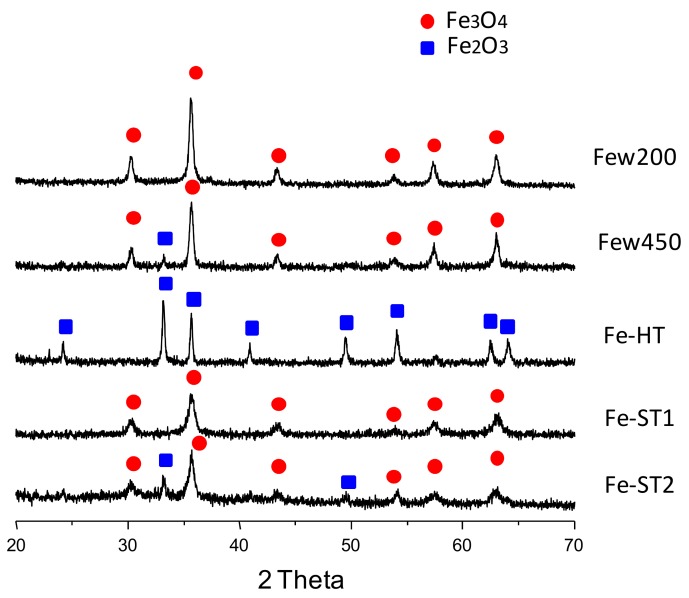
XRD patterns of the iron oxide catalysts synthesized. Crystalline phases: ● Fe_3_O_4_ (spinel-type magnetite, JCPDS: 19-629, or maghemite, JCPDS No.39-1346) and ■ Fe_2_O_3_ (JCPDS: 24-0072).

Figure 3 shows the XRD patterns for the prepared catalysts. The diffraction maxima obtained permit unambiguous identification of which structure type is stabilized in each case, corundum Fe_2_O_3_ and/or spinel-type Fe_3_O_4_. All the iron catalysts show the spinel-type structure as the main crystalline phase, except the hematite-type phase (corundum-type Fe_2_O_3_) observed in the case of the Fe-HT sample. However, whilst the only identified phase for Few200 and Fe-ST1 catalyst is spinel-type Fe_3_O_4_, the other two catalysts prepared, Few450 and Fe-ST2, also present the Fe_2_O_3_ phase as a minority.

**Figure 4:**
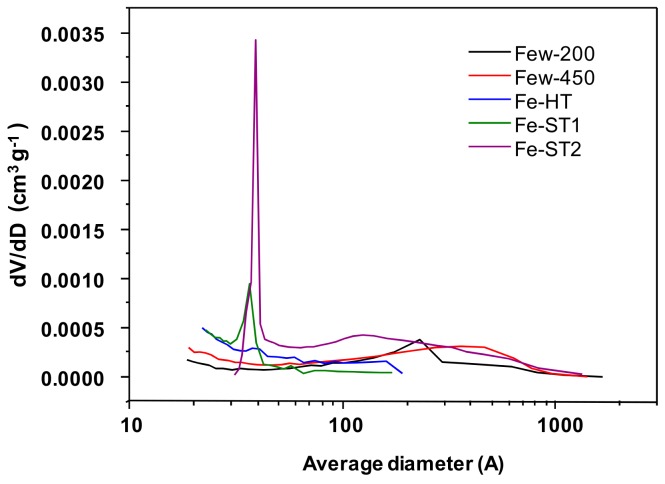
Pore-size distributions for the prepared catalysts.

Textural characteristics of the samples were analyzed by N_2_ adsorption isotherm measurements. The main textural data are gathered in Table 2 and the corresponding pore size distributions are shown in Figure 4. It can be seen that the surface area varies from 18 m^2^ g^−1^ in Fe-ST1 catalyst to 55 m^2^ g^−1^ for Fe-ST2 catalyst. Interestingly, Few200, Few450, Fe-ST1 and Fe-HT show a similar surface area of around 25 m^2^ g^−1^. It is noteworthy that the samples obtained under supercritical water conditions (Few200 and Few450) present very high maxima mesopore diameters as well as adsorption average pore diameters (D4V/A-Meso), likely probing the presence of interparticle mesoporosity among FeOx nanocrystallites. On the other hand, it is worth pointing out that the Fe-ST2 sample presents a bimodal distribution with a narrow and intense peak centered at about 4 nm, and a broad peak centered at 15 nm (Figure 4). Surprisingly, as shown later by HRTEM, both peaks can be related to the presence of interparticle mesoporosity, surface cavities and pores, respectively. Besides, the Fe-ST1 and Fe-HT samples only present porosity in the 2–6 nm range, although less prominent than for the Fe-ST2 sample. This type of mesoporosity could be related to the presence of different amounts of open cavities in the iron oxide nanoparticles.

Through the easy preparation methods used in the present article, we can tune up the main iron oxide crystalline phases formed. Interestingly, different catalysts (Few200, Few450, Fe-ST1 and Fe-HT) with similar surface areas, ca. 25 m^2^ g^−1^, but different characteristics can be prepared. This way, a more accurate catalytic discussion regarding the influence of the nature of the iron oxide properties will be possible.

**Figure 5:**
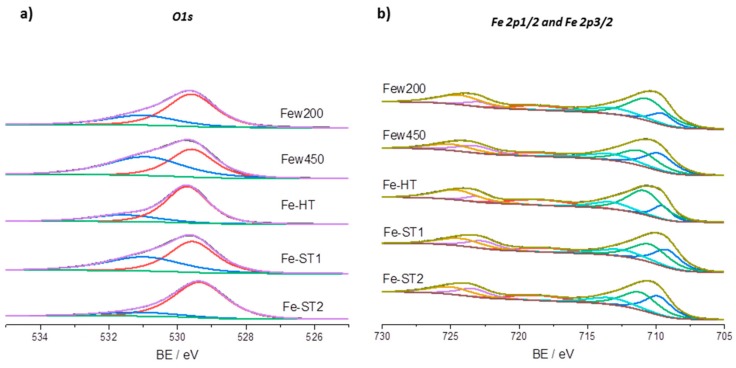
XPS spectra for the differently prepared FeOx catalysts. (**a**) O1s and (**b**) Fe 2p_1/2_ and Fe 2p_3/2_.

In order to know the surface characteristics of these catalysts, XPS experiments were carried out (Figure 5 and Table 3).

It must be noted that the position of the peaks of Fe 2p_1/2_ and Fe 2p_3/2_ as well as their satellite peaks are highly sensitive to the iron oxidation state in our catalysts. Fe 2p photoelectron peaks are at around 710.7 eV and 724.3 eV, presenting a shake-up satellite at 718.7 eV, which is ca. 8 eV above the BE of Fe 2p_3/2_. The gap of the 2p doublet is ca. 13.6 eV. All this corresponds to characteristic features of Fe^3+^ [24,25]. The satellite feature at 718.7 eV is clearly observed for all these samples. This satellite is most pronounced for pure Fe_2_O_3_, whereas it is not or hardly observed for pure Fe_3_O_4_ [26,27,28]. Overall, both Fe^2+^ and Fe^3+^ have been observed in all the catalysts but in order to quantify their relative amounts a deconvolution of Fe 2p peaks is necessary. Besides the predominant Fe^2+^ and Fe^3+^ peaks, a higher BE energy shoulder was also fitted around 713.4 eV, which may be related to an interaction between Fe^2+^ and Fe^3+^, being similar to the reported interaction between Fe core and Fe_2_O_3_ shell [29]. Because the shoulder at 713.4 eV has a considerably large weight in the area of the measured envelope and is well mixed with contributions from the sub-peaks at 709.9 eV (Fe^2+^) and 711.2 eV (Fe^3+^), it is difficult to obtain the exact Fe^2+^/Fe^3+^ ratios on the surface of the samples from the recorded XPS spectra. Nevertheless, a tentative deconvolution was carried out for the different samples, The evaluation of the surface Fe^2+^/Fe^3+^ ratios from the XPS deconvolution indicates that the Fe^2+^/Fe^3+^ ratio on the surface of the nanoparticle samples is the lowest in the Fe-HT catalyst, as expected, since the only crystalline phase detected by XRD in this sample is Fe_2_O_3_, a phase where iron is present only as Fe^3+^. In the other catalysts, in which Fe_3_O_4_ is majority, the proportion of Fe^2+^ is accordingly higher (44–53%).

The characteristics of surface O-species were also evaluated by XPS. Thus, the O1s peaks are deconvoluted into two different peaks at binding energies of about 529.6 and 531 eV, suggesting the presence of O with two distinct chemical environments. The assignment of the oxygen species is not straightforward. The bands at binding energies of ca. 529 eV, called as O_α_, can be related to the presence of surface lattice oxygen (O^2−^) whereas the BE at 531–532 eV, named as O_β_, is due to the presence of defect oxide or to surface low coordination oxygen ion. It has also been reported that some contribution to the 531–533 eV peak proceeds from carbonates or surface hydroxyl species [30,31]. Catalysts synthesized by the hydrothermal method or solvothermal method with PEG (Fe-HT and Fe-ST2 catalysts) present a higher proportion of lattice O_α_ species (73–79%) than the other catalysts. Conversely, the highest contribution of O_β_ species, which are thought to be beneficial to the Mars–van-Krevelen mechanism for VOCs oxidation, is found for those iron nanoparticles prepared in supercritical water by continuous regime (46 and 55% of O_β_).

**Figure 6:**
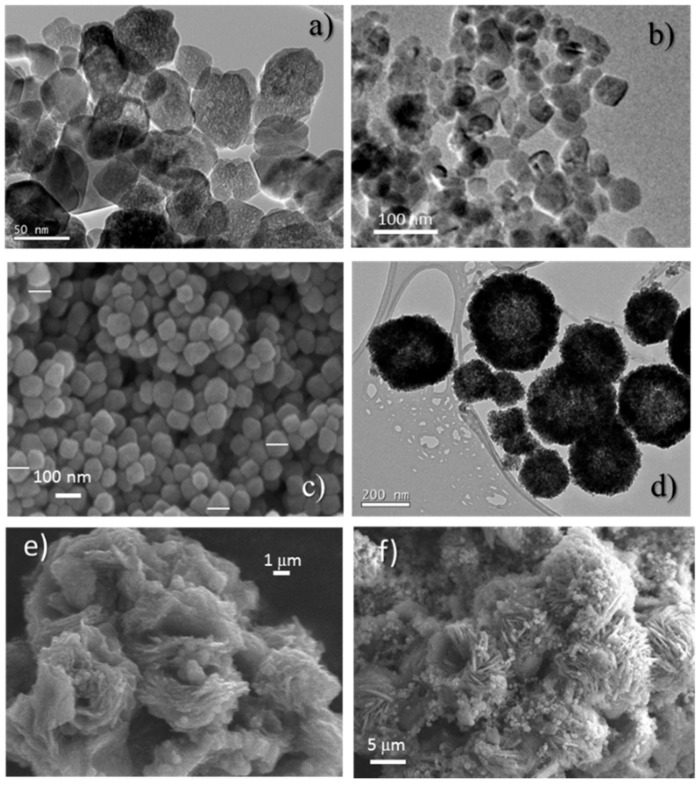
Representative EM images for the (**a**) Few200, (**b**) Few450, (**c**) Fe-HT, (**d**) Fe-ST1 and (**e**) Fe-ST2 fresh catalysts and (**f**) iron glycolate precursor of Fe-ST2.

The microstructure of the samples was analyzed by microscopy. Several representative images of the catalysts are shown in Figure 6. The samples obtained in supercritical water present well-defined pseudospherical nanocrystals with different sizes depending on the particular catalyst synthesized. The spheres observed in the sample treated at lower temperature, Few200, are in the 7–45 nm range whereas the sample treated at higher temperature, Few450, presents larger spheres of about 10–70 nm diameter. For both samples, inner cavities are observed. According to N_2_ adsorption data, it can be assumed that internal cavities are not connected to the nanocrystal surface. Therefore, only interparticle mesoporosity is mainly obtained.

In contrast, the Fe-ST1, Fe-ST2 and Fe-HT samples are composed of greater nanoparticles. In the Fe-HT catalyst large spheres of 60–100 nm were mainly observed, whereas in the Fe-ST1 catalyst blackberry-type aggregates of ca. 200 nm, composed of small units of about 6 nm are clearly visible. Finally, the Fe-ST2 sample presents spherical particles of ~100 nm embedded in flower-like formations of ~10 μm. This morphology results from the decomposition of the glycolate precursor, which develops first, before calcination (Figure 6f).

**Figure 7:**
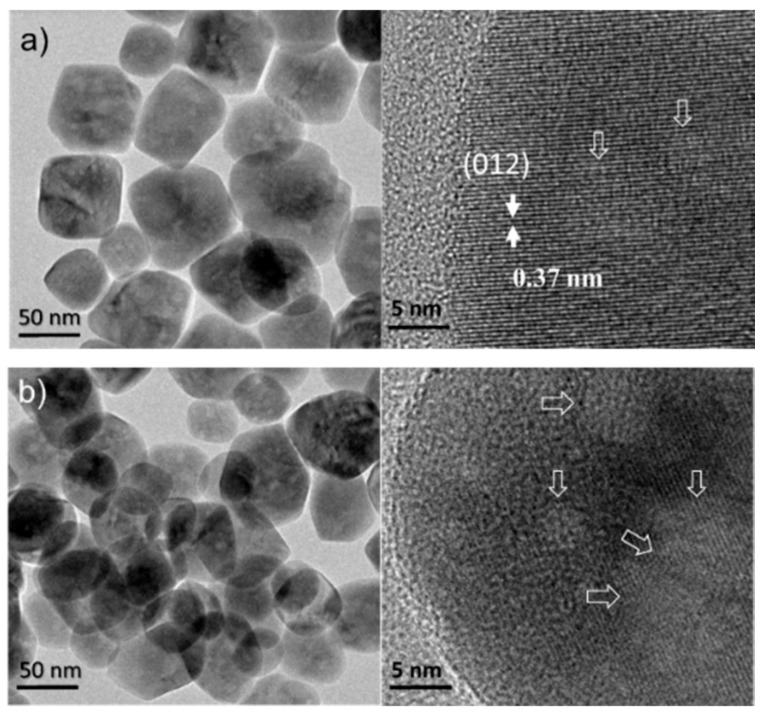
High-resolution transmission electron microscopy (HRTEM) images of the Fe-HT catalyst (**a**) before use and (**b**) after three catalytic cycles. Arrows indicate nanocavities.

As Fe-HT and Fe-ST2 catalysts present the best catalytic performances, they have been characterized before and after reaction by high-resolution HRTEM. As previously mentioned for both samples, a stable catalytic performance has been observed. The fresh Fe-HT catalyst (Figure 7a) presents a homogeneous distribution of single crystalline spongy spheres of about 60–100 nm. Some nanocavities are clearly appreciable, which actually are flat sheets of ca. 6 nm. This morphology is coherent with the average pore diameter close to 6 nm as stated from textural measurements (see Table 2), indicating that the porosity corresponds to open cavities. After three catalytic cycles (Figure 7b), the Fe-HT material shows similar particles which are now slightly more agglomerated (this could be related to sample history, too). In this case, slightly greater nanocavities are observed, a few of them of up to 15 nm in diameter. In spite of the subtle morphological differences observed, the catalytic performance has remained stable (not shown here) after 3 cycles.

**Figure 8:**
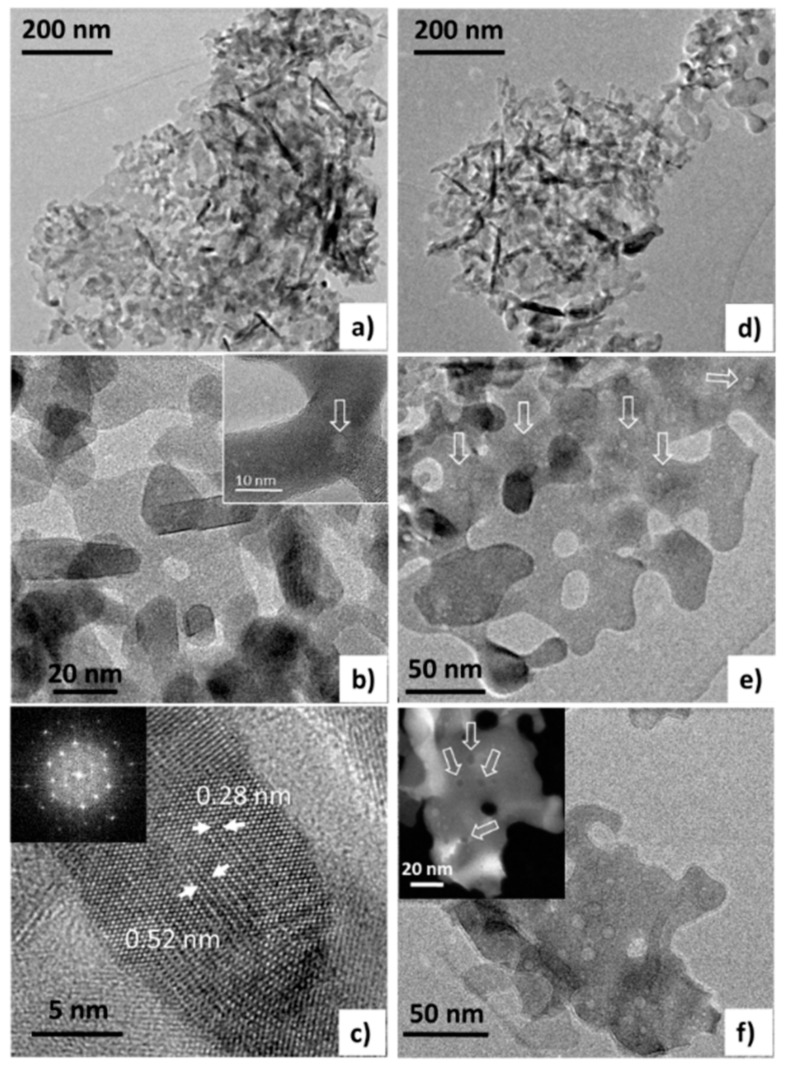
HRTEM images at different magnifications of the Fe-ST2 catalyst (**a**–**c**) before use and (**d**–**f**) after three catalytic cycles. Unfilled arrows indicate nanocavities. Insets: in (**c**) fast Fourier transform (FFT) coherent with indicated distances, which correspond to (111) and (220) interplanar distances of spinel structure; in (**f**) corresponding Scanning Transmission Electron Microscopy (STEM) image, in which nanocavities are clearly appreciated as black zones.

On the other hand, the Fe-ST2 catalyst (Figure 8a–c) shows an irregular morphology consisting of plate-like particles of nanometric thickness and stick-like particles. Plate-like formations of low crystallinity present holes in the middle of about 15–20 nm (Figure 8a,b), showing a ring-like appearance. These holes provide additional intraparticle porosity to the sample, likely increasing the number of accessible active sites. This morphology suggests the template role of the iron glycolate, as mentioned above (see Figure 6e,f). High-magnification images could be interpreted considering spinel-type structure, in concordance with XRD data (Figure 3c). Additionally, surface cavities are clearly identified (Figure 8b). In line with N_2_ adsorption data, open cavities with a mean diameter size about 5 nm are available at the catalyst surface. After three cycles (Figure 8d–f), the Fe-ST2 catalyst shows particles with similar morphology but presenting a greater number of nanocavities of 2–10 nm. Thus, in spite of these subtle changes no variation has been observed in the catalytic activity after several runs.

## 4. Discussion

**Figure 9:**
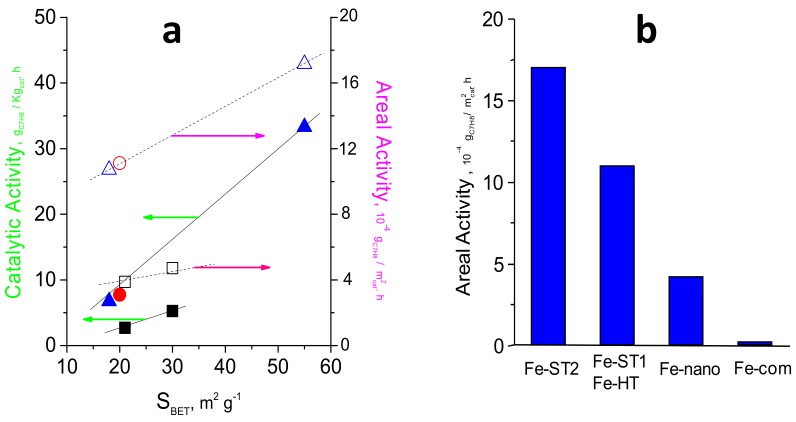
(**a**) Influence of the preparation method and the surface area of the FeOx prepared catalysts on the catalytic activity per gram of catalyst and on the catalytic activity per surface area in the toluene oxidation. (**b**) compares the values of the areal activity for the most efficient catalysts prepared in the present work with those of a high surface area Fe-nano and the commercial Fe-com. Note: 275 °C, GHSV = 300,000 h^−1^ and remaining reaction conditions detailed in the text. Symbols: Open symbols correspond to catalytic activity per surface area. Filled symbols correspond to catalytic activity per gram of catalyst. (∆, ▲) solvothermal method, (○, ●) hydrothermal method, (□, ■) supercritical water method.

All these catalysts yield a relevant catalytic activity for the total oxidation of toluene. Figure 9a shows the influence of the surface area on the catalytic activity. It can be observed that, in general, the catalytic activity increases with the surface area of the catalyst although the preparation method also plays an important role. Then, those catalysts prepared using subcritical conditions, either through solvothermal or hydrothermal methods, are more active than those synthesized using supercritical water. As seen in Figure 4, Figure 5, Figure 6, Figure 7 and Figure 8, a different type of cavities could explain this behavior. Whilst only interparticle porosity is observed in the case of the samples prepared under supercritical conditions, pointing out the presence of internal cavities, some narrow intraparticle porosity is attained for those samples prepared under subcritical conditions (open cavities), leading to more active surface sites. Accordingly, if we consider now the catalytic activity normalized per surface area, it can also be observed that solvothermal or hydrothermal methods present the best values. Moreover, the two samples prepared by the solvothermal method have active sites with different activity, being the intrinsic activity of Fe-ST2, 50% higher than that found for Fe-ST1. A different amount of open cavities on the catalyst surface could explain this behavior. In this sense, regardless of the bulk structure in each case, the catalytic activity seems to be significantly affected by the availability and activity of active sites, e.g., defectively bonded Fe cations at the surface which became more or less available mainly depending on the morphological features of the different samples.

In order to benchmark the catalytic performance of these catalysts, Figure 9b shows a comparison of the catalytic activity normalized per surface area between the optimal catalysts synthesized in the present work and two reference catalyst: a high surface area iron oxide prepared by a nanocasting route (Fe-nano) and a commercial iron oxide (Fe-com). Fe-ST2 displays the highest areal activity (ca. 1.7 × 10^−3^ g_C7H8_/m^2^ h) with Fe-ST1, and Fe-HT presenting similar activity (in the 1.05–1.15 × 10^−3^ g_C7H8_/m^2^ h range). These values are higher than that achieved by a high surface area, Fe-nano, catalyst prepared by a nanocasting route (ca. 4 × 10^−4^ g_C7H8_/m^2^ h) and remarkably higher than the commercial iron oxide, Fe-com. Again, surface area does not seem to be the only factor controlling the observed response.

Since the surface area value is not the solely determinant factor, the behavior of samples with similar area may allow appropriate comparison of the intrinsic reactivity of the active sites. Therefore, we try to compare the performance of those catalysts (Few200, Few450, Fe-HT and Fe-ST1) that present similar areas in the 18–30 m^2^ g^−1^ range. In these catalysts, different crystalline phases and different morphologies can be observed. Firstly, Few200 and Fe-ST1 only present as a crystalline phase, according to XRD, Fe_3_O_4_, and no apparent differences in the preferential planes exposed are observed. However, Fe-ST1 is remarkably more active than the Few200 catalyst so that we have to consider other factors in order to understand this behavior. It can be observed that the morphological properties are quite different as Fe-ST1 is formed by large aggregates composed of small units (ca. 6 nm) with appreciable interparticle pores and some open cavities, whereas Few200 is formed by small and medium (7–45 nm) spherical nanoparticles with internal cavities. XRD patterns also confirm the presence of different size nanocrystals since the peaks of Fe-ST1 are notoriously wider than those of Few200. Therefore, although the presence of different types of cavities could have a preponderant influence on the intrinsic activity of the active sites, it cannot be ruled out that small crystallites could also lead to higher catalytic activity. Finally, it should be considered the proportion of structural defects obtained from XPS data. Again, it can be seen how the characteristics of the catalyst surface are playing an important role in the catalytic reaction. According to XPS data, although the relative amounts of Fe^2+^ are higher than the theoretical values for both Fe_3_O_4_ catalysts (in the spynel-type Fe_3_O_4_ phase around 67% must be Fe^3+^ and 33% of Fe^2+^), it can be observed that surface Fe^2+^ for Fe-ST1 is significantly higher than that found in Few200, suggesting that the presence of a high concentration of oxygen surface vacancies could be an important parameter for toluene activation at lower temperature [6]. Therefore, it is worth commenting that although the total oxidation of hydrocarbons on metal oxides has often been reported to take place through a redox Mars–van-Krevelen mechanism, in which the surface-adsorbed oxygen species play an essential role, this could not be true for this catalytic system. Accordingly, no relationship has been found between the amount of O_β_ species (detected by XPS) and the catalytic activity for these two Fe_3_O_4_ catalysts. Indeed, the catalyst with the lowest areal activity, Few200, shows the highest concentration of surface-adsorbed oxygen species. These results seem to point out that the mechanism of the total oxidation of toluene on iron oxide catalysts might proceed through an Elay–Rideal mechanism in which the role of the oxygen surface vacancies is predominant. The existence of structural defects favors the adsorption of molecular oxygen, leading to very active oxygen species and, in turn, improving the catalytic activity for toluene total oxidation. Therefore, the characteristics of the surface and the morphology should be the responsible for this different performance. On the other hand, the influence of the crystalline phase could be evaluated comparing the catalytic activity of Few200 and Few450 samples. These catalysts have comparable textural, surface and morphological properties. However, a higher intrinsic catalytic activity is observed in the case of the Few450 sample. This behavior could be tentatively related to the presence of Fe_2_O_3_ as a minor phase where, in line with XPS data, the relative amount of oxygen surface vacancies is greatly increased. In line with the previous statements, if the catalytic performance of Fe-HT and Fe-ST1 catalysts are compared, we can observe similar values. Whilst Fe-HT is composed of Fe_2_O_3_ spheres with marginal open cavities and a relevant amount of oxygen surface vacancies detected as surface Fe^2+^, Fe-ST1 consists of Fe_3_O_4_, but with smaller mean crystallite size and higher content of open cavities. Thus, these effects seem to be counterbalanced, leading to catalysts with comparable catalytic activity.

Herein, we have observed that oxygen vacancies seem to be the active sites for toluene activation. Accordingly, the surface area and the relative amount of oxygen defects for VOCs adsorption would be key parameters as more adsorption sites would be available whereas the reducibility would not contribute so much. Additionally, as shown in Figure 9b, the intrinsic activity of these active sites might be dependent on the catalyst morphology since those catalysts with internal open cavities present the highest areal rate. On the contrary, surface-adsorbed oxygen species do not seem to be involved in the toluene’s total oxidation, since the catalyst with the highest areal activity, Fe-ST2, shows the lowest concentration. This fact might be related to the presence of cavities in the iron oxide nanocrystals produced after the mild preparation treatments.

One important point to take into account is the possible role of hydroxyl and carbonate groups in the catalytic performance. In the case of the hydroxyl groups as all our samples have been calcined up to 400 °C before use, the hydroxide-type phases seem to be absent according to the thermogravimetric analysis (TGA) curves and Fourier transform–infrared (FT–IR) spectra (Supporting Information, Figure S1). The FT-IR spectra before and after reaction show two peaks at 1639 and 3400 cm^−1^, which can be assigned to the vibration bending and stretching modes of O–H, indicating the presence of adsorbed water on the surface of the sample. The peaks at 780 and 910 cm^−1^ can be related to the in-plane bending of surface hydroxyl groups, i.e., Fe–OH–Fe. In addition, the band at 3150 cm^−1^ is related to the stretching mode of Fe–O–OH in α-FeOOH. These bands almost disappear (they present a clear decrease in intensity) after the use in the catalytic reaction. These hydroxyl surface species could act as toluene adsorption sites for further activation by molecular oxygen adsorbed at the oxygen surface vacancies. However, in-situ Diffuse Reflectance Infrared Fourier Transform Spectroscopy (DRIFTS) measurements should be carried out in order to bring some light into this assumption.

Regarding to the presence of carbonates, some additional peaks are noticed in the used catalysts compared to the fresh catalyst: a band at 2330 cm^−1^, attributed to the asymmetric stretching of adsorbed molecular CO_2_ and another one at 1036 cm^−1^ assigned to the (C–O) symmetric vibrational mode of carbonates. These peaks demonstrate the C-deposition during the toluene oxidation. Therefore, carbonates are indeed formed after use. Fortunately, the stable catalytic performance of these catalysts can suggest that the formation of carbonates after use does not result in a deteriorated catalytic behavior.

In the iron oxide catalysts studied, the active sites, e.g., defectively bonded Fe cations, became more or less active and preponderant mainly depending on the morphological features of the different samples. Then, although all these catalysts yield a relevant catalytic activity for the total oxidation of toluene, the highest specific and intrinsic catalytic activity observed in the iron oxides tested in the present work corresponds to Fe-ST2 catalyst, which is characterized by the highest surface area (55 m^2^ g^−1^), the highest mesopore volume (about 0.25 cm^3^ g^−1^) including 10–20 nm internal mesopores, a low mean crystallite size calculated from the Scherrer equation (about 15 nm), a relevant proportion of oxygen surface defects (about 80%), the presence of two crystalline phases—Spinel-type Fe_3_O_4_ (main) and hematite Fe_2_O_3_ (minority)—and the occurrence of a bimodal pore size distribution with a significant amount of open surface cavities. All these parameters seem to be relevant to obtain a catalyst with remarkable activity for toluene total oxidation.

## 5. Conclusions

Cavity-containing porous iron oxides have been synthesized by mild routes. These oxides show high and stable reactivity in the total oxidation of toluene. The preparation method has been decisive in achieving high reactivities, since both surface area and the type of cavity-containing nanoparticles are different. Whilst internal cavities are obtained under supercritical water conditions, open cavities are attained using subcritical preparation methods, especially under solvothermal conditions. The presence of these cavities has different positive effects on the catalyst performance depending on their nature. Whilst internal cavities only seem to increase the amount of oxygen vacancies, open cavities also increase the intrinsic activity of the active sites. Hence, the catalyst displaying the best performance, Fe-ST2, presents a morphology mainly consisting of holed quasi-bidimensional plates with internal cavities, which remain almost unchanged after cycling.

Interestingly, the nature of the crystalline phase also seems to modify the catalytic performance. Thus, for catalysts prepared using either subcritical or supercritical conditions, the catalysts with the highest activity are those which present some Fe_2_O_3_ apart from Fe_3_O_4_ (Few450 and Fe-ST2) whereas those with only Fe_3_O_4_ (Few200 and Fe-ST1) are less active. A higher surface percentage of defectively bonded Fe cations is always observed when the Fe_2_O_3_ minor crystalline phase appears. From the catalytic viewpoint, the stability of these samples is a very interesting aspect to highlight as no variation in the catalytic activity in the toluene oxidation has been observed after several cycles. The presence of water in the feed has been shown to be negative for the catalytic activity. However, the drop in the activity is not drastic and the stability observed after several cycles working with water is excellent. The two most active catalysts, Fe-HT and Fe-ST2, were characterized after use in the reaction, showing very subtle differences with respect to the fresh catalysts, which did not have any impact on the catalytic performance.

## Figures and Tables

**Table 1:** Summary of notation, preparation and some physicochemical properties of the FeOx samples.

Catalyst	Synthesis Method	S_BET_ (m^2^ g^−1^)	Morphology and Size ^a^	Fe-Oxide Phases ^b^
Few200	In supercritical water. Continuous regime. T = 200 °C (subcritical value)	21	Spherical φ ~7–45 nm	Fe_3_O_4_ spinel
Few450	In supercritical water. Continuous regime. T = 450 °C (supercritical value)	30	Spherical φ ~10–70 nm	Fe_3_O_4_ >> Fe_2_O_3_
Fe-HT	Hydrothermal in subcritical conditions. Batch regime (sealed autoclave)	20	Spherical with cavities φ ~30–100 nm	Fe_2_O_3_ corundum
Fe-ST1	Solvothermal in subcritical conditions. Ethylene glycol Batch regime (sealed autoclave)	18	Blackberry aggregates 200 units (~6 nm)	Fe_3_O_4_ spinel
Fe-ST2	Solvothermal in subcritical conditions. Polyethylene glycol in ethylene glycol. Batch regime (sealed autoclave)	55	Flower-like aggregates (~10 μm)	Fe_3_O_4_ >> Fe_2_O_3_

^a^ Determined by electron microscopy (EM). ^b^ Crystalline phases observed by X-ray diffraction (XRD) and confirmed by EM.

**Table 2:** Textural characteristics of the iron oxide catalysts synthesized.

Sample	S_BET_ (m^2^ g^−1^)	V_Meso_ (cm^3^ g^−1^)	D_Meso_ (Å)	D_4V/A-Meso_ (Å)
Few200	21	0.14	231.1	236.5
Few450	30	0.21	362.1	248.3
Fe-HT	20	0.03	39.5	61.4
Fe-ST1	18	0.02	36.5	42.5
Fe-ST2	55	0.26	38.5	204.2

**Table 3:** X-ray photoelectron spectroscopy (XPS) results of the iron oxide catalysts synthesized (Oα and Fe^2+^ ratios given in percentage).

Catalyst	Oxygen Signals Detected O1s			Iron Signals Detected (eV)		
	O_α_ (eV)	O_β_ (eV)	O_α_/O	2p_3/2_	Satellite	Fe^2+^/(Fe^2+^ + Fe^3+^)
Few200	529.5	531.0	54	710.6	718.7	34
Few450	529.6	530.9	45	710.7	718.8	49
Fe-HT	529.8	531.6	73	710.7	718.8	22
Fe-ST1	529.6	531.0	62	710.7	718.8	44
Fe-ST2	529.3	531.1	79	710.7	718.9	45

## Supplementary Materials

The following are available online at http://www.mdpi.com/1996-1944/11/8/1387/s1, Figure S1: TGA and FTIR spectra of fresh and used Fe-ST1 catalyst.
