# Supplementary material for: Eco-Friendly Cavity-Containing Iron Oxides Prepared by Mild Routes as Very Efficient Catalysts for the Total Oxidation of VOCs
Source: Materials (Basel). 2018 Aug 9;11(8):1387. doi: 10.3390/ma11081387 (PMC6119960; doi:10.3390/ma11081387)
Supplement: Supplementary file 1 [file materials-11-01387-s001.pdf]

Supporting Information

Eco-friendly cavity-containing iron oxides prepared by mild routes as very efficient catalysts for the total oxidation of VOCs

Rut Sanchis [1], Daniel Alonso-Domínguez [2], Ana Dejoz [1], María Pilar Pico [3], Inmaculada Álvarez-Serrano [2], Tomás García [4], María Luisa López [2,*], Benjamín Solsona [1,*]

[1] Departament d'Enginyeria Química, ETSE, Universitat de València, Av. Universitat, 46100, Burjassot, Valencia, Spain.
[2] Departamento de Química Inorgánica, Facultad de Ciencias Químicas, Universidad Complutense de Madrid.
[3] Sepiolsa, Avda. del Acero, 14-16, Pol. UP-1 (Miralcampo), 19200-Azuqueca de Henares, Guadalajara, Spain.
[4] Instituto de Carboquímica (CSIC), C/Miguel Luesma 4, 50018 Zaragoza, Spain.

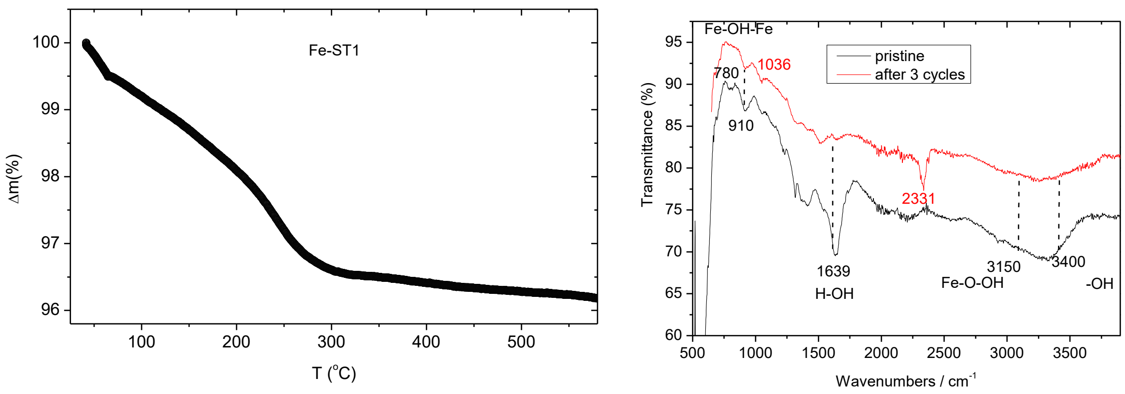

Figure S1. TGA and FTIR spectra of fresh and used Fe-ST1 catalyst.
